# Medical and Surgical History of the War of the Rebellion

**Published:** 1873-04

**Authors:** 


*Medical and Surgical History of The War of the Rebellion.* Prepared in Accordance with Acts of Congress, under the direction of Surgeon-General JOSEPH K. BARNES, U. S. A. Part first, two volumes. Vol. I Medical, Vol. II Surgical. Washington: Government Printing Office.

The lessons which war tesches are sad and terrible, but none the less valuable.

The American people in the dreadful school of a four year's civil war have learned themselves and have demonstrated to the other nations of the earth, many valuable truths. Among the many lessons which we have of necessity learned is one whose value can never be estimated and whose practical importance has been duly appreciated by foreign powers, the care of the sick and wounded soldier. The medical staff of the United States Army, when the civil war first opened was in many respects undeveloped. The comparative quiet of the Amerienn army for the long time preceeding the Rebellion had made the duties of the army surgeon nearly nominal, and he was in many instances without the least experience in the care of the wounded or in the construction or management of army hospitals.

The indefatigable skill and industry displayed by the Surgeon General and his assistants in developing the medical organization of the Army, deserves the highest praise. That they should find time in the labor necessary to organize the medical corps to devote to the collection of statistics and scientific facts is a matter of wonder, and evinces their complete devotion to the cause scientific medicine.

The collection of the data for this History was commenced under the direction of Surgeon-General HAMMOND, who worked many excellent reforms during his brief term of service, and has been ably continued under the careful supervision of Surgeon-General BARNES, his successor.

The first volume is devoted to the medical portion of the work, and is edited by Surgeon J. J. WOODWARD. The volume opens with a preface by the Surgeon-General which gives a history of the work and its preparation, this is followed by an introduction by the editor.

The statistical tables are formed on a modification of the classification advocated by Dr. FARR. To these tables seven hundred and twenty-six pages are devoted.

The appendix comprises over three hundred pages and consists of Reports of Medical Officers and extracts from the returns of different Surgeons, and is a very interesting portion of the medical literature of the war.

The Surgical Volume is compiled under direction of Surgeon GEO. H. OTIS, who gives an introduction which will be read by all with interest and profit.

To dispel the idea that the post of Army Surgeon is one that is free from danger, a list of medical officers who lost their lives or were wounded in discharge of duty is given, it comprises nineteen who were killed in action, thirteen who were killed by partizan troops or assassinated by guerrillas or rioters, eight who died of wounds received, nine who died from accident while in discharge of their duty, and seventy-three who were wounded in battle. This large record speaks in no feeble terms of the bravery and devotion to duty which characterized the medical staff of the United States Army

The surgical volume is handsomely illustrated by cuts of specimens, illustrations of wounds, etc., etc.: this volume is devoted merely to wounds of the head and chest, those of the abdomen and pelvis, together with some remarks upon injuries to the head and spine being reserved for the succeeding volume. The two volumes which have been issued speak well for the care and diligence displayed by those having them in charge. They are a contribution to medical science which should cause the profession to feel proud of the brethren of the army medical staff.

